# An effective correlation-based data modeling framework for automatic diabetes prediction using machine and deep learning techniques

**DOI:** 10.1186/s12859-023-05488-6

**Published:** 2023-10-02

**Authors:** Kiran Kumar Patro, Jaya Prakash Allam, Umamaheswararao Sanapala, Chaitanya Kumar Marpu, Nagwan Abdel Samee, Maali Alabdulhafith, Pawel Plawiak

**Affiliations:** 1 Department of ECE, Aditya Institute of Technology and Management, Tekkali, AP 532201 India; 2 School of Computer Science and Engineering, VIT Vellore, Katpadi, Vellore, Tamil Nadu 632014 India; 3 Department of Information Technology, College of Computer and Information Sciences, Princess Nourah bint Abdulrahman University, P.O. Box 84428, Riyadh, 11671 Saudi Arabia; 4 Department of Computer Science, Faculty of Computer Science and Telecommunications, Cracow University of Technology, Warszawska 24, 31-155 Krakow, Poland; 5 Institute of Theoretical and Applied Informatics, Polish Academy of Sciences, Bałtycka 5, 44-100 Gliwice, Poland

**Keywords:** Diabetes, Correlation, Deep learning, CNN, Health care, PIMA Indian diabetes, Machine learning

## Abstract

The rising risk of diabetes, particularly in emerging countries, highlights the importance of early detection. Manual prediction can be a challenging task, leading to the need for automatic approaches. The major challenge with biomedical datasets is data scarcity. Biomedical data is often difficult to obtain in large quantities, which can limit the ability to train deep learning models effectively. Biomedical data can be noisy and inconsistent, which can make it difficult to train accurate models. To overcome the above-mentioned challenges, this work presents a new framework for data modeling that is based on correlation measures between features and can be used to process data effectively for predicting diabetes. The standard, publicly available Pima Indians Medical Diabetes (PIMA) dataset is utilized to verify the effectiveness of the proposed techniques. Experiments using the PIMA dataset showed that the proposed data modeling method improved the accuracy of machine learning models by an average of 9%, with deep convolutional neural network models achieving an accuracy of 96.13%. Overall, this study demonstrates the effectiveness of the proposed strategy in the early and reliable prediction of diabetes.

## Introduction

Diabetes is a chronic health condition that affects millions of people worldwide. It is characterized by high levels of sugar (glucose) in the blood, which can lead to serious health complications if left untreated [1, 2]. There are two main types of diabetes: type 1 and type 2. Type 1 diabetes, also known as juvenile diabetes, is an autoimmune disorder in which the body’s immune system attacks and destroys the cells that produce insulin, a hormone that regulates blood sugar levels. Type 2 diabetes, on the other hand, is a metabolic disorder caused by a combination of genetic and lifestyle factors, such as obesity and lack of physical activity [3]. It is the most common type of diabetes, accounting for about 90–95% of all cases. People with type 2 diabetes either do not produce enough insulin or their bodies are resistant to it.

Diabetes can lead to a variety of serious health complications, including heart disease, stroke, kidney disease, diabetic retinopathy [4], and amputations [5]. It also increases the risk of developing certain cancers, such as endometrial, breast and colon cancer. However, with proper management and treatment, people with diabetes can live long and healthy lives. Treatment for diabetes typically includes monitoring blood sugar levels, making healthy lifestyle choices, and taking medications or insulin as needed. According to statistics, 463 million people globally have diabetes in 2019, with the number expected to rise to 578 million by 2030 and 720 million by 2045. As a result, the number of diabetic patients is expected to rise exponentially by 25% in 2030 and 51% in 2045 [6].

At present, early diagnosis of diabetes is performed manually by a physician doctor based on his or her expertise, experience, and observation of the condition. The healthcare industry currently gathers a large quantity of data, but this data may not necessarily disclose inherited hidden patterns, as is the case with genetic data. These manual judgments are, therefore, extremely deceptive and harmful, especially in the case of an early diagnosis, because some factors may be overlooked, resulting in a severe influence on the observations and consequences [7]. It is difficult to make accurate predictions about the onset of diabetes. However, while there is no protracted treatment for diabetes, it can be treated and managed if a correct diagnosis can be made early in the disease’s progression. Moreover, early diagnosis of diabetes can help to avoid complications and reduce the likelihood of developing serious health problems. Thus, sophisticated early and automated diagnostic procedures are urgently needed to improve accuracy.

There are several machine-learning techniques that can be used for diabetic prediction, including:

- Logistic Regression: This is a statistical method that can be used to predict the probability of a binary outcome, such as whether or not a patient has diabetes.
- Decision Trees: This method involves creating a tree-like model of decisions and their possible consequences, with the goal of predicting the outcome of a new patient based on their characteristics.
- Random Forest (RF): This is an ensemble method that combines the predictions of multiple decision trees to improve the overall accuracy of the model.
- Support Vector Machine (SVM): This is a supervised learning algorithm that can be used for classification or regression problems.
- Neural Networks: This is a set of algorithms, modeled loosely after the human brain, that is designed to recognize patterns. They can be used for a variety of tasks, including diabetic prediction.
- Gradient Boosting algorithm: This is an ensemble method that combines the predictions of multiple decision trees to improve the overall accuracy of the model.

These are some of the most popular machine-learning techniques that can be used for diabetic prediction. Still, it is important to note that the choice of technique will depend on the specific characteristics of the data and the goals of the analysis. All the above machine-learning techniques are dependent on manual feature extraction. Hence deep learning techniques came into existence for the purpose of classification with automatic feature extraction, but it is important to note that the choice of technique will depend on the specific characteristics of the data and the goals of the analysis.

The primary contributions of this work can be summarized as follows:

- To increase the effectiveness of the classification method, we introduced a unique novel data modeling technique and integrated it with a Deep Convolutional Neural Network for making accurate predictions about the onset of diabetes.
- The proposed framework employs a pre-processing phase to get rid of duplicates, inconsistencies, missing values, and outliers for better understanding.
- A reliable training strategy, such as 5-fold cross-validation, was performed to increase the method’s universal effectiveness and minimize over-fitting.
- The suggested data modeling framework improves performance and convergence time compared to other traditional methods in the literature.
- Statistical analysis is utilized to validate the significance of the data modeling technique and that is recommended for use with various classifiers.
- To show the proposed system’s effectiveness, we compare it to numerous state-of-the-art methods using various evaluation metrics.

The remaining of the paper is structured as follows: related works are discussed in Sects. 1, and 2 provides materials and methods such as dataset information, pre-processing, and a complete framework for data modeling. Section 3 presents the experimental simulation, findings, and performance indicators. Section 5 contains detailed discussions on potential work with various techniques and Statistical analysis. Finally, Sect. 4 concludes the paper with a conclusion.

## Related works

In response to the rising diabetes epidemic, various artificial intelligence (AI) techniques have been developed to find hidden patterns in huge healthcare data sets. In recent years, various machine learning and deep learning frameworks for diabetes prediction have been presented [8–11]. Some researchers implemented diabetes prediction using ML techniques such as Artificial Neural Networks (ANN) [12], SVM [13, 14], Naïve Bayes [15], Linear Discriminant Analysis (LDA) [15], Nearest Neighbor (NN) [16] and RF [17] by utilizing a variety of dimensionality reduction and cross-validation approaches. Kumar *et al.* [18] used various classification techniques, including SVM, ANN, and classification tree, to predict type-2 diabetes and obtained an accuracy ranging between 73.00% and 80.00%. The major risk factors for developing type 2 diabetes were analysed by Miah et al. [4]. More important parameters related to type-2 diabetes and its effects on QoL were identified using the technique of correlation analysis. The authors of [19] evaluated the efficacy of well-known machine learning approaches (ANN, K-NN, and decision trees) for diabetes mellitus prediction. Experiments were conducted on two databases, one obtained from a Frankfurt hospital and the other from an open-source PIMA Indian dataset. The results indicated that the best overall accuracy was 90.00

In addition, Tafa et al. [20] came up with a model that uses SVM and Naive Bayes together to predict diabetes. A set of data from three different places in Kosovo was used to test the model. The dataset includes 8 key attributes, and 80 of 402 people in the study had type 2 diabetes. In order to perform the validation test, they partitioned the dataset, so that half (50%) of it was used for the training set and the other half for the testing set. The authors reported that the accuracy of the SVM was 95.50%, while the accuracy of the Naive Bayes classifier was 90.00%. An ANN model presented by authors in [21] can be highly valuable for healthcare officials and practitioners. The author was prompted by the disease’s extremely deadly complication. They designed an ANN model for reducing the training error function. Therefore, the determined average error function was 0.01%, and the accuracy achieved by ANN was 87.30%. Soltani et al. [22] proposed a diabetic prediction system using Probabilistic Neural Network (PNN). The experiment was conducted using the Pima Indians Medical Diabetes (PIMA), and the data was split between 90% training and 10% testing. The proposed network achieved an overall training accuracy of 89.50% and a testing accuracy of 82.00%. Using factors such as sleep, routine, food, exercise, insulin, and heart rate, Rodrguez et al. [23] used feature selection on diabetes (type 1) patients. For each feature, the authors used time-series data and the Sequential Input Selection Algorithm (SISAL) to rank the importance of the feature in relation to its predictive value for blood glucose levels.

Deep learning has achieved significant advances in data processing [24], computer vision [25–27], and some other applications [28–32]. In recent years, experts have started recognising DL methods’ potential for handling massive datasets [24]. Consequently, diabetes prediction has also been accomplished utilizing DL methods. Deep Neural Networks were utilised for the study by Ashiquzzaman et al. [33]. The architecture of the DNN is made of the Multi-layer Perceptron (MLP), the General Regression Neural Network (GRNN), and the Radial Basis Function (RBF). The PIMA data set served as the basis for evaluating the method. The dataset is divided so that 192 samples are used for the testing set, while the remaining samples are used for the training set. The authors claimed that their findings were accurate 88.40% of the time. Further, Swapna et al. [34] employed two DL approaches to increase diabetes prediction accuracy. Electrocardiograms were utilised to evaluate the performance of CNN and CNN-LSTM using a private dataset. Using five-fold cross-validation, the dataset was divided into training and testing sets. Both models achieved an accuracy of 90.90% and 95.10% after being constructed. The Recurrent Neural Network (RNN) was utilised by Ramesh et al. [35] in order to make a prediction regarding the two forms of diabetes. The Pima Indian dataset, which consists of 768 samples and eight features, was employed by the authors. In order to verify the results of the study, the dataset was divided so that 80% would be used for training and 20% would be used for testing. The accuracy of diabetes type-1 prediction was 80.60%, whereas the accuracy of diabetes type-2 prediction was 75%. The many methods of diagnosing diabetes are outlined in Table 1, which provides a summary of these methods.

The performance of the existing methods is limited to 80% in diabetic prediction. The main drawback is the availability of diabetic data is very less, but deep learning techniques require large data for better performance. Hence, the creation of synthetic data is required that looks similar to the original data but with different variations. This can help to improve the performance of a model by providing it with more diverse training data, which can make the model more robust and less prone to over-fitting. Data augmentation techniques for one-dimensional data involve transforming the existing data in various ways to create new, synthetic data that can be used to expand the original dataset. Some examples of data augmentation techniques for one-dimensional data include:

- Addition of noise: Random noise can be added to the data to simulate real-world variations.
- Scaling: The data can be scaled up or down to create variations in the magnitude of the values.
- Rotation: The data can be rotated around a certain point to create new variations.
- Time shifting: The data can be shifted forward or backwards in time to create new variations.
- Interpolation: New points can be interpolated between existing data points to create new data.
- Re-sampling: The data can be re-sampled by reducing or increasing its frequency to create new variations.
- Reverse: Reversing the data can be useful in some cases.

All the above traditional approaches are not able to provide better results. Therefore, feature-based correlation techniques are proposed to overcome the problems in the existing literature.

**Table 1:** Similar approaches for diabetic prediction using PIMA dataset

**Author (s)**	**Techniques**	**Result**	**Remarks**
Kandha Swamy et al. [36]	Multiple ML based algorithms: SVM, K-NN, J48 and Random Forest	73.82% with J48 classifier and claimed 100% with K-NN	There is no adequate explanation is provided for the pre-processing procedure that was performed on the dataset.
Yuvraj *et al.* [37]	Random Forest, Decision Tree and Naïve Bayes classifier with data processing	Claimed 94% and 84% accuracies with Random Forest Classifier and Decision Tree	Not specified how the data was pre-processed, although they did outline the Information Gain approach for feature selection, which was utilized to extract the important features.
Sisodia et al. [38]	Decision Tree, Naïve Bayes and SVM approach with Data Pre-processing.	Reported highest accuracy of 76.30% with Naïve Bayes	Experimentation was carried out with 10 fold cross-validation, and there was no more clear information on data processing.
Olaniyi et al. [39]	Multi Layer Feed Forward Network (MLP-NN)	Reported 82% accuracy with MLP-NN	Before processing the data for classification, the authors normalized the dataset in order to get a stable numerical representation.
Ashiquzzaman et al. [33]	Deep Neural Networks with MLP, GRNN, and RBF	Claimed an accuracy of 88.41%	The authors made a conscious decision not to pre-process the dataset because DNN is capable of filtering the data and acquiring the biases.
Zhou et al. [40]	Enhanced Deep Neural Network	Reported an accuracy of 94.02%	Model is primarily designed with the help of a deep neural network’s hidden layers and it make use of dropout regula- -rization in order to avoid over-fitting.
Yahyaoui et al. [41]	Convolutional Neural Network	Reported an accuracy of 76.81%	TThere is no adequate information on methodology and techniques.
Naz et al. [42]	Decision Tree and Naive Bayes	96.62% and 76.33 % Accuracies reported	The authors worked on different classifiers and reported accuracies in the range between 76% to 97%.
Abdulhadi et al. [43]	Random Forest Classifier	Reported an accuracy of 82%	TThere is no adequate information on data pre-processing and methods.
Abdollahi et al. [44]	Ada boost algorithm	Reported an accuracy of 92%	This study aimed for integraion of different data mining techniques and developed ensemble based training to improve the performance.

Motivated by the earlier discussion, this article proposes a comprehensive data modeling framework for early diabetes prediction. Initial interest in data modeling originates from the correlation between attributes and outcomes. Furthermore, a number of ML-based algorithms were used to carry out the classification process. In addition, a deep CNN network is used with a data-modeling strategy for enhanced performance and substantial outcomes. PIMA dataset features were used for training the entire model. The proposed model can also help doctors make more accurate decisions for diabetes screening and early diagnosis based on the quality of the data.

## Materials and methods

**Fig. 1:**
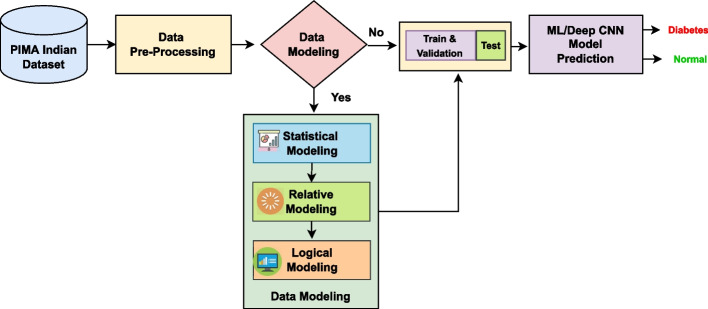
Proposed methodology for diabetes prediction

The proposed work mostly focused on developing a data modeling framework with the intention of giving more relevant data to the input of the learning algorithm for the purpose of making an early prediction of type-2 diabetes among individuals. The proposed work is implemented in five main stages: Dataset preparation, data Pre-processing, data modeling framework, data splitting, and classification (using Ml/customized DL), presented in Fig. 1.

### Dataset preparation

Several past investigations [45] have generated publicly available datasets for diabetes prediction and diagnosis. On the other hand, it has been shown that the fatality rate of diabetes is higher in women than in men. This is due to the fact that the number of fatalities connected with diabetes in 2019 is 2.3 million for women and 1.9 million for men [46]. The PIMA Indian dataset was utilized for this work, and it was obtained from the UCI Machine Learning repository (Available at https://www.kaggle.com/datasets/uciml/pima-indians-diabetes-database). This data was provided by the NIDDK [34], which is part of the National Institute of Diabetes and Digestive and Kidney Diseases. The PIMA dataset included 768 females over 21 years old, 500 of whom were diabetic negative and 268 of whom were diabetic positive. The dataset has been widely used to estimate the risk of diabetes for each given observation based on the eight most influential independent features. This dataset is one of the most flexible, benchmarked, and trustworthy diabetes prediction datasets. Table 2 offers a thorough overview of the dataset attributes.

**Table 2:** Detailed overview of PIMA dataset features

**S. No**	**Selected feature**	**Details**	**Range**	**Average**
1	Pregnancies (F1)	The frequency with which a woman gives birth	0-17	3.85
2	Glucose (F2)	Plasma glucose levels at 2 hours in a glucose tolerance test administered orally	0-199	120.90
3	Blood Pressure (F3)	Diastolic blood pressure (when blood flows into the arteries that connect the heart) (mm Hg)	0-122	69.11
4	Skin Thickness (F4)	The thickness of the triceps skin fold (mm)	0-99	20.54
5	Insulin (F5)	Insulin concentration in serum throughout a 2-hour time period (mu U/ml)	0-846	79.81
6	BMI (F6)	Body mass index (weight in kg/(height in m^2^)	0-67.1	31.99
7	Diabetes Pedigree Function (F7)	The function that calculates diabetes risk based on family history	0.08-2.42	0.47
8	Age (F8)	The participants age in years	21-81	33.24
9	Outcome (Label)	Diabetes class variable *Yes* means that the patient has diabetes, and *No* means that the patient doesn’t have diabetes.	Yes/No	Yes/No

### Data pre-processing

The quality of the data is essential since it has such a significant impact on the accuracy and reliability of the predictions [34]. The pre-processing stage deals with null/unknown values in the data and excludes any outliers. The refined data set was utilized to form a prediction model. Before applying classifiers to the data index, the data should be appropriately pre-processed and organized. Before moving on to the next step, this data should be kept in good shape for better results. The dataset contains some missing values and null values in the subsequent attributes. Features with null or unknown values are replaced with zero; however, some features, such as F2 and F3, may not be zero. In addition, missing values are substituted by calculating the mean of the attribute associated with the target (outcome). Finally, the feature values were rescaled in order to produce the typical normal distribution with a zero mean and unit variance. Equation (1) is beneficial for such attributes with missing values.

1 $$\begin{aligned} MV({F_i}) = \,\left\{ \begin{array}{l} mean\,({F_i})\,\,\,\,\,if\,F\,is\,missing/null\\ {F_i}\,\,\,\,\,\,\,\,\,\,\,\,\,\,\,\,\,\,\,\,\,\,\,\,\,\,\forall \,i = 1\,to\,8 \end{array} \right. \end{aligned}$$

Where MV($F_i$) represents the missing value and $F_i$ denotes feature value ranging from *i*=1 to 8.

### Data modeling approach

The process of developing a significant data flow for the purpose of managing the data and achieving the desired results is referred to as data modeling. A comprehensive data model assists in developing a streamlined, logical database that removes redundancy and allows for efficient retrieval. In this work, the objective of the data modeling technique is to contribute appropriate attributes to the input of artificial intelligence-based prediction algorithms (ML/Deep CNN), with the eventual goal of achieving positive performance. The technique that has been suggested is carried out in three steps, which include statistical modeling, relative modeling, and logical modeling respectively.

### Statistical modeling

A mathematical representation of the observed data is referred to as a statistical model. The technique of applying statistical analysis to a dataset is known as statistical modeling. In statistical analysis, one of the helpful components is called a parameter. It is a term that refers to the qualities that are utilized in the process of defining a certain value. In this investigation, the three well-known statistical parameters mean, median and variance stated in Eq. (2)-(3) were applied to each of the eight characteristics. After data pre-processing, the proposed statistical parameters for the PIMA dataset were computed and given in Table 3.

2 $$\begin{aligned} Mean({{{\bar{F}}}_i})= & {} \,\frac{{\sum \limits _{j = 1}^k {{F_i}(j)} }}{N} \end{aligned}$$

3 $$\begin{aligned} Standard\,Deviation\,({\sigma _j})\,= & {} \,\sqrt{\frac{{{{({F_j} - \,{{{\bar{F}}}_i})}^2}}}{N}} \end{aligned}$$

Where *i*= Number of the features/ attributes ranging from 1 to 8 that is *F1* to *F8*. Where *k* and *N* are the total number of instances (*N*=*k*=768) and *j*= sample size ranging from 1 to *k*.

**Table 3:** Statistical parameters calculation

**S. No**	**Feature/Attribute**	**Statistical features before and after pre-processing**					
		**Mean** **(Before)**	**Mean** **(After)**	**Median** **(Before)**	**Median** **(After)**	**Standard ** **Deviation** **(Before)**	**Standard** **Deviation** **(After)**
1	Pregnancies (F1)	3.8450	3.8241	3	3	3.3695	3.3241
2	Glucose (F2)	120.8945	119.121	117	114	31.9726	29.8172
3	Blood Pressure (F3)	69.1054	71.145	72	71	19.3558	12.3150
4	Skin Thickness (F4)	20.5364	26.652	23	26	15.9522	9.5851
5	Insulin (F5)	79.7994	79.5741	30.5	30.56	115.244	76.2182
6	BMI (F6)	31.9925	32.1942	32	32	7.8841	6.6210
7	Diabetes Pedigree Function (F7)	0.4718	0.3560	0.3725	0.36	0.3313	0.2749
8	Age (F8)	33.2408	32.7162	29	29	11.7602	11.1864

**Fig. 2:**
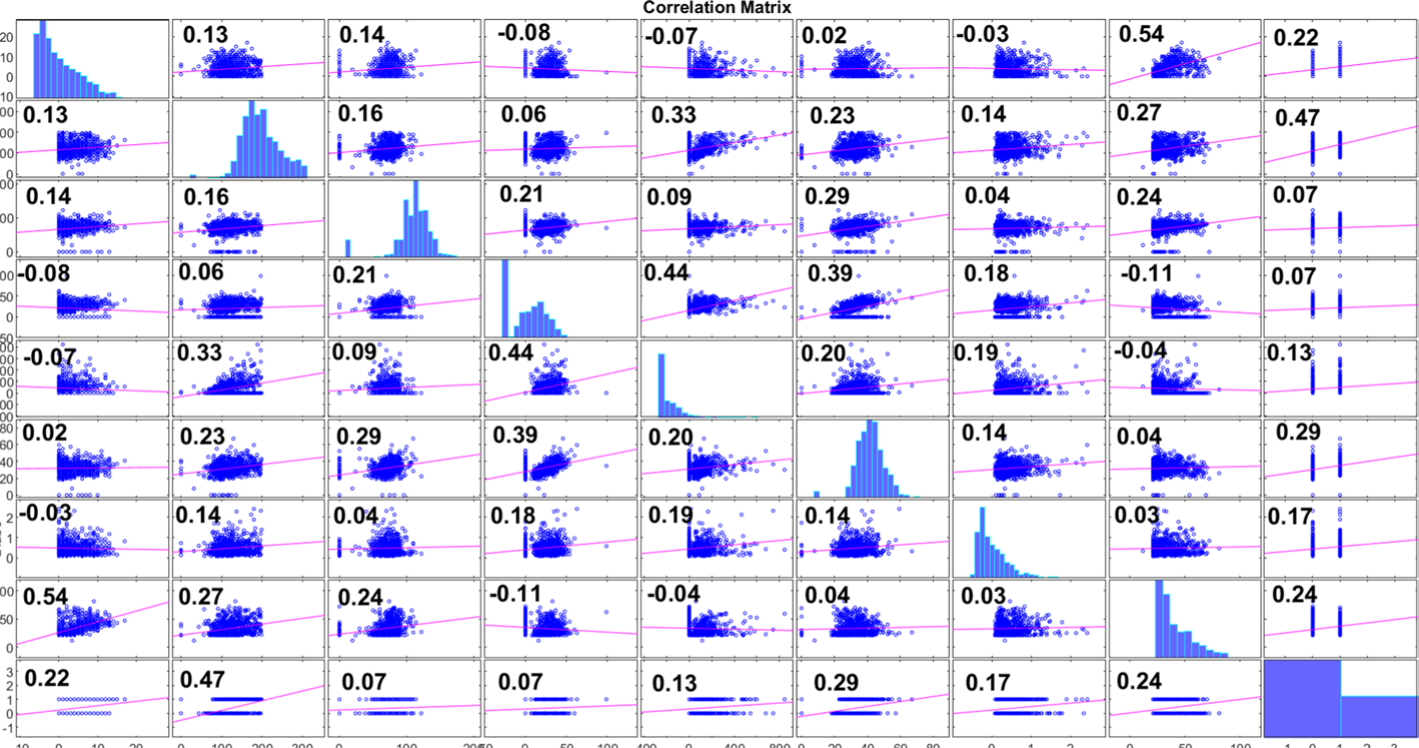
Correlation Matrix for the dataset

### Relative modelling

Relative modeling is a method that is primarily focused on the variation of a particular attribute in relation to the statistical measure that corresponds to it. This method will provide a comprehensive understanding of the pre-processed dataset. The relative modeling strategy is taken into consideration in this study based on the correlation measure that exists between the features and the outcome. However, only features with a high degree of correlation should be chosen for further processing in the relative modeling strategy. The correlation plot between the different aspects of the PIMA Indian dataset, as well as the features’ relationships with the outcomes, is illustrated in Fig. 2. According to the correlation plot, among all of the features, the glucose (*F2*) feature has a high correlation (0.47). In contrast, the features of blood pressure (F3) and skin thickness (*F4*) have attained a low correlation (0.07). The features with significant correlation, such as glucose (*F2*), BMI (*F6*), age (*F8*) and pregnancies (*F1*), will be considered for relative modeling. The suggested relative variation is computed as follows:

4 $$\begin{aligned} M{V_R}\,({F_i})\,= & {} \,\left| {{F_j} - {\bar{F}}} \right| \end{aligned}$$

5 $$\begin{aligned} M{D_R}\,({F_i})\,= & {} \,\left| {{F_j} - (F{M_i})} \right| \end{aligned}$$

6 $$\begin{aligned} S{D_R}\,({F_i})\,= & {} \,\left| {{F_j} - {\sigma _i}} \right| \end{aligned}$$

Where *i*=2, 6, 8 and *j*= 1 to *N* (*N*= sample size=768), whereas(${{\bar{F}}}$), $FM_i$ and $\sigma _i$ are the mean, median and Standard deviation of the corresponding pre-processed feature. $MV_R$, $MD_R$ and $SD_R$ stand for the relative value of the mean, median and standard deviation, respectively. All the values in this approach are considered absolute values and processed to the next stage. Some of the sample features (*F2* and *F6*) for the reference are reported in Table 4. After this approach, the size of the input dataset is quite large for the experimentation, and the feature dimension increased from 768$\times$8 to 768$\times$20.

**Table 4:** Features after relative approach

**Feature**	**Feature value** **(pre-processed)**	**Relative value ** **of mean** **(MVR)**	**Relative value ** **of median** **(MDR)**	**Relative value of ** **Standard Deviation** **(SDR)**
Glucose (F2)	148	28.879	34	118.1828
	85	34.121	29	55.1828
	183	63.879	69	153.1828
	89	30.121	25	59.1828
	137	17.879	23	107.1828
	116	3.121	2	86.1828
BMI (F6)	33.6	1.4058	1.6	26.979
	26.6	5.5942	5.4	19.979
	23.3	8.8942	8.7	16.679
	28.1	4.0942	3.9	21.479
	43.1	10.9058	11.1	36.479
	25.6	6.5942	6.4	18.979
	33.6	1.4058	1.6	26.979

### Logical modeling

In order to improve the efficiency of the machine learning system, the features that were generated during the relative modeling phase are passed on to the logical modeling phase. The logical modeling technique binarized certain attributes based on specified criteria. This approach mostly focuses on converting the nominal values of relative attributes into binary attributes. Based on certain parameters shown in Eqs. (7)–(9), the refined features from the relative model approach are encoded into binary ones and zeros.

7 $$\begin{aligned} LFM({F_i})= & {} \,\left\{ \begin{array}{l} 1,\,\,\,\,\,\,If\,M{V_R}({F_i}) > 0\\ 0,\,\,\,\,\,\,Otherwise \end{array} \right. \end{aligned}$$

8 $$\begin{aligned} LFMD({F_i})= & {} \,\left\{ \begin{array}{l} 1,\,\,\,\,\,\,If\,M{D_R}({F_i}) > 0\\ 0,\,\,\,\,\,\,Otherwise \end{array} \right. \end{aligned}$$

9 $$\begin{aligned} LFSD({F_i})= & {} \,\left\{ \begin{array}{l} 1,\,\,\,\,\,\,If\,S{D_R}({F_i}) > \,\,\mathop {S{D_R}({F_i})}\limits ^{\_\_\_\_\_\_\_\_\_\_} \\ 0,\,\,\,\,\,\,Otherwise \end{array} \right. \end{aligned}$$

Where *i*=2, 6, and 8 were the recommended attributes based on the relative approach. However, LFM($F_i$), LFMD($F_i$) and LFSD($F_i$) refer to logical features that are based on the mean, the median, and the standard deviation, respectively. Following the completion of the logical phase processing, the input dataset dimension was extended even further to 768$\times$32, and the results of certain sample features are shown in Table 5.

**Table 5:** Features after logical approach

**Feature**	**Feature ** **value** **(pre** **-processed)**	**Relative ** **value ** **of mean** **(MVR)**	**Logic ** **Feature** **(LFM)**	**Relative** **value ** **of median** **(MDR)**	**Logic ** **Feature ** **(LFMD)**	**Relative ** **value ** **of Standard** **Deviation** **(SDR)**	**Logic ** **Feature ** **(LFSD)**
Glucose (F2)	148	28.879	1	34	1	118.1828	1
	85	34.121	0	29	0	55.1828	0
	183	63.879	1	69	1	153.1828	1
	89	30.121	0	25	0	59.1828	0
	137	17.879	1	23	1	107.1828	1
	116	3.121	0	2	1	86.1828	0
BMI (F6)	33.6	1.4058	1	1.6	1	26.979	1
	26.6	5.5942	0	5.4	0	19.979	0
	23.3	8.8942	0	8.7	0	16.679	0
	28.1	4.0942	0	3.9	0	21.479	0
	43.1	10.9058	1	11.1	1	36.479	1
	25.6	6.5942	0	6.4	0	18.979	0

## Experimental results

The experimental results of the recommended methods for the early detection of diabetes are provided in this section. In our suggested method, we utilize the PIMA Indian dataset and apply it to several AI-based methods. Two types of tests are conducted with each classifier on the input dataset. In the initial experiment, all eight original features were employed, and the size of the input dataset was 768 x 8. In the second experiment, we suggested a data modeling approach to improve the quality and quantity of features, consequently improving the performance of the prediction system. The preliminary stages of the proposed work are processed in MATLAB 2021b environment, and later on Python programming language with compact deep learning libraries Keras and Tensorflow is utilised for testing. All of the proposed codes have been run on a system with an Intel *i7* processor, 16 GB of *DDR3*, and an *NVIDIA RTX 2060* graphics card. Experimentation with machine learning algorithms and deep learning algorithms is discussed in Sects. 3.1 and 3.2, respectively.

### Machine learning classifiers performance

In this work, significant well-known machine learning classifiers such as MLP-NN, SVM, and RF were used for diabetic prediction. Initially, each classifier was applied to the original PIMA Indian dataset with eight features, and performance parameters were calculated. In the subsequent experiment, each classifier was evaluated with the 768 x 32 redesigned dataset derived from the data modeling strategy. In all experiments, the entire dataset was divided into 80% for training cum validation and the remaining 20% for testing the model. We used 5-fold cross-validation for model assessment and its statistical performance. The training process is used to train a model, which is subsequently utilized in the testing process to determine the model’s efficiency.

**Fig. 3:**
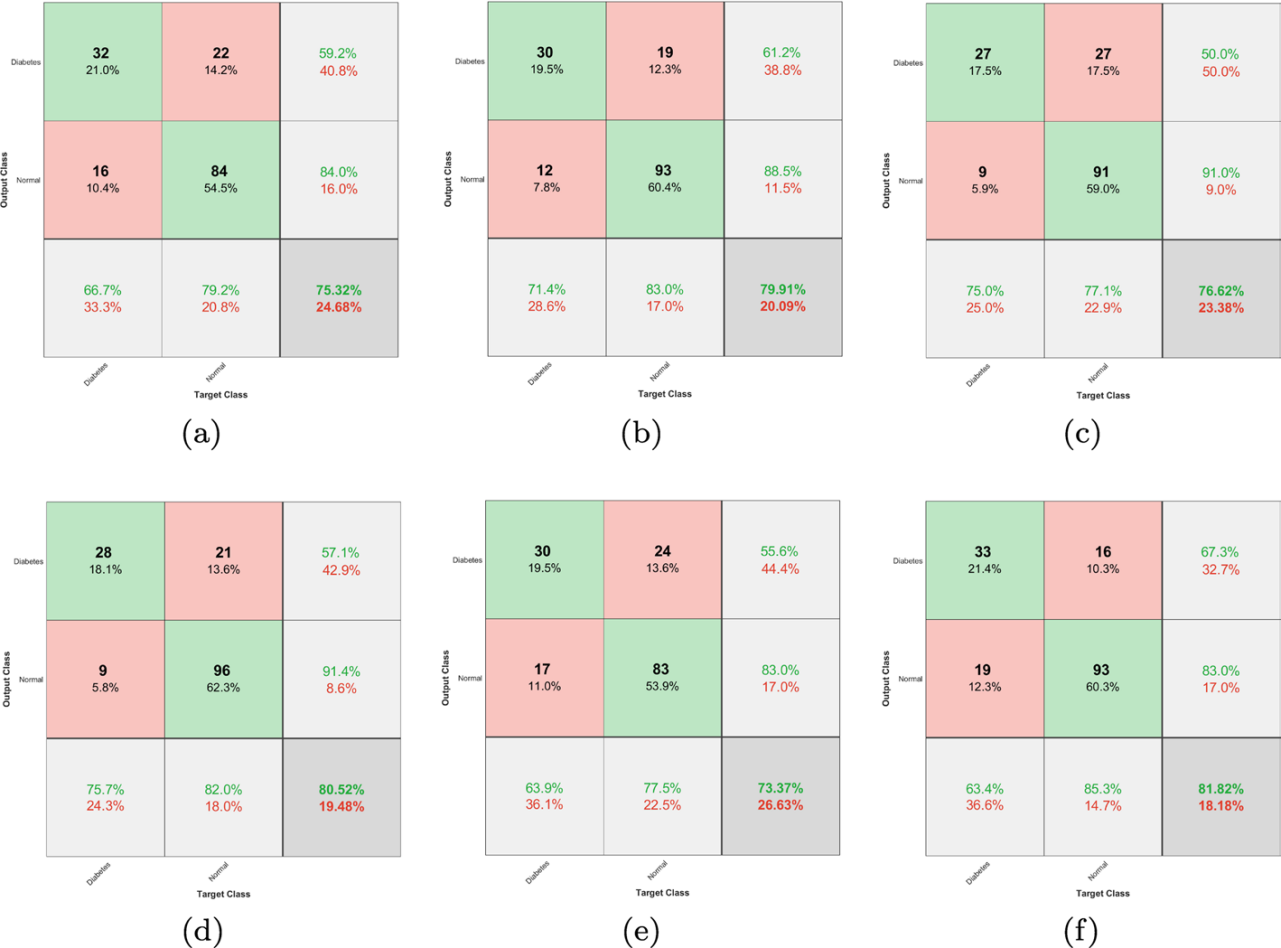
Confusion Matrices generated from ML Classifiers for Diabetic Prediction

The Fig. 3 illustrates the confusion matrix obtained by the simulation results for suggested machine learning classifiers (MLP-NN, SVM and RF) with and without data modeling. The resulting confusion matrix only has two classes: diabetes and normal. Each column in the confusion matrix represents the actual class, and each row represents the predicted class. To evaluate the performance of each classifier, we employed typically recognized performance measures, including accuracy, precision, recall, F-Measure, and ROC area [47]. The performance metrics are reported in Eqs. (10)–(14).

10 $$\begin{aligned} {\textrm{Accuracy}}= & {} \frac{\mathrm{TP + TN}}{\mathrm{TP + TN + FP + FN}} \end{aligned}$$

11 $$\begin{aligned} {\textrm{Sensitivity}}= & {} \frac{{ \mathrm TP}}{\mathrm{TP + FN}} \end{aligned}$$

12 $$\begin{aligned} {\textrm{Specificity}}= & {} \frac{{ \mathrm TN}}{\mathrm{TN + FP}} \end{aligned}$$

13 $$\begin{aligned} {\textrm{Precision}}= & {} \frac{\textrm{TP }}{\mathrm{TP + FP}} \end{aligned}$$

14 $$\begin{aligned} {\mathrm{F{-}Measure}}= & {} \frac{{2*\Pr ecsion*{\mathop {\textrm{Re}}\nolimits } call}}{{\Pr ecision + {\mathop {\textrm{Re}}\nolimits } call}} \end{aligned}$$

Where *TP*= True Positive, *TN*= True Negative, *FP*=False Positive, and *FN*=False Negative, and all these are calculated from the Confusion Matrix.

**Fig. 4:**
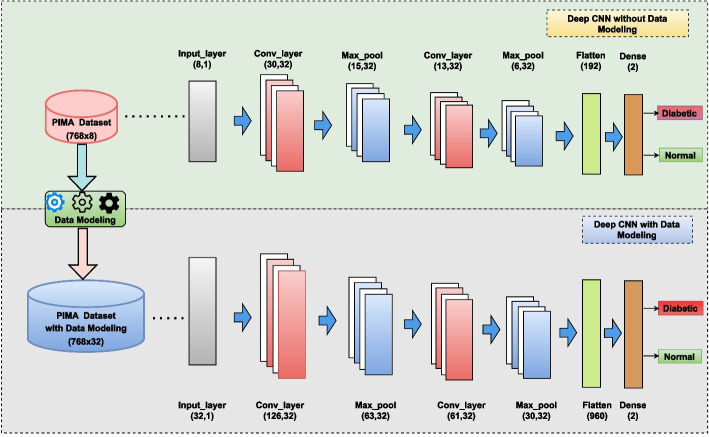
Deep CNN architecture for diabetes prediction

The performance parameters for the classifiers with and without data modeling are reported in Table 6. All these parameters were computed using the confusion matrix generated from each classifier. Compared to the original dataset, it has been observed that the input dataset using the data modeling techniques significantly improves the performance of the recommended ML models. The MLP-NN model resulted in an increase in the accuracy of predictions from 75.32 to 79.87%. In addition, the accuracy of the predictions made by the SVM model improved from 76.62 to 80.52%. The RF classifier also enhanced the test’s accuracy, increasing it from 73.37 to 82.82%. Overall, the proposed data modeling strategy enhanced the accuracy of all the suggested ML models by an average of 9%.

**Table 6:** Performance evaluation for ML classifiers

**Technique(s)**	**Parameter**	**MLP-NN**	**SVM**	**RF**
Without data modeling (Only eight features)	Accuracy (%)	75.32	76.62	73.37
	Precision (%)	74.81	76.41	72.84
	Recall (%)	75.33	76.66	73.42
	F-Measure (%)	75.0	75.36	72.91
	ROC Area	0.784	0.705	0.786
With data modeling (enhanced features)	Accuracy (%)	79.87	80.52	82.82
	Precision (%)	79.37	80.0	81.59
	Recall (%)	79.99	80.55	81.88
	F-Measure (%)	79.41	79.97	81.65
	ROC Area	0.845	0.783	0.855

### Performance of the deep learning model

The accuracy that was attained in this study by applying classic machine learning algorithms like MLP-NN, SVM, and RF was inadequate. The findings obtained through the use of ML classifiers cannot be validated as a strategic tool for the early-stage prediction of diabetes. In the proposed work, we utilize the data modeling approach on the PIMA Indian dataset and then apply it to a customized deep learning network. Furthermore, it can assist healthcare professionals in making better decisions based on data features. The details of the proposed deep learning network architecture are presented in Fig. 4. There were a total of 768 instances in the dataset, and this work split them as follows: 80% (614 instances) were used for training and validation, and the remaining 20% (154 instances) were used for testing the model. We used 5-fold cross-validation for model assessment and its statistical performance. Table 7 provides the mean values for the indicators used in the 5-fold cross-validation.

**Fig. 5:**
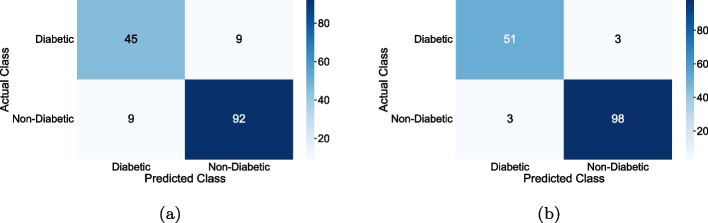
Confusion Matrices Generated from Deep CNN for Diabetic Prediction

**Table 7:** Performance indicators for 5-fold cross-validation

**Parameter**	**Fold-1**	**Fold-2**	**Fold-3**	**Fold-4**	**Fold-5**	**Avg.**±**SD**
Accuracy (%)	97.51	97.28	97.68	95.94	97.49	97.181±0.707
Sensitivity (%)	95.38	96.55	94.41	96.82	95.59	95.754±0.967
Precision (%)	96.21	94.47	95.38	96.18	96.71	95.791±0.878
Specificity (%)	98.74	96.88	97.18	97.51	98.11	97.680±0.746
F-Measure (%)	95.55	96.12	94.46	95.84	95.42	95.473±0.630

**Fig. 6:**
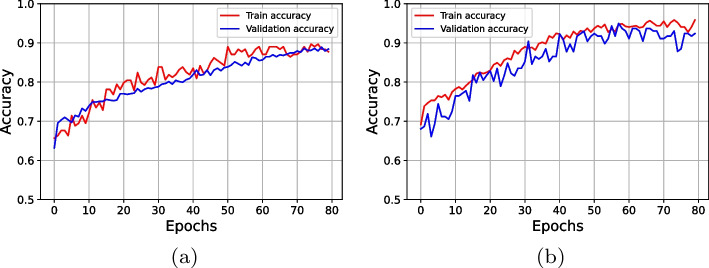
Training and validation accuracy for diabetes prediction

**Fig. 7:**
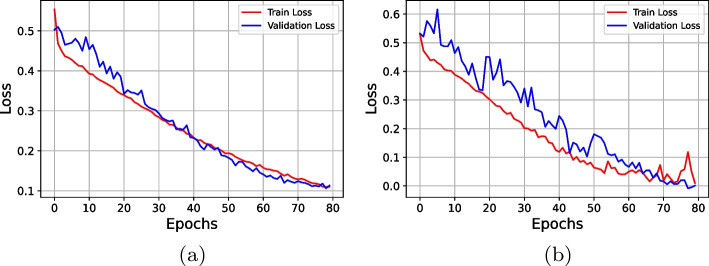
Training and validation loss for diabetes prediction

**Fig. 8:**
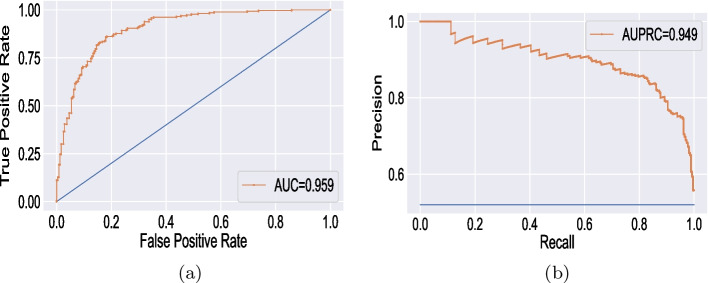
Classification performance curves for diabetes prediction

The learning rate, the number of epochs, and batch size are the most crucial variables for customizing the CNN model, and these values determine how well the suggested deep CNN model performs. In this work, after executing several experiments, the best prediction performance for the CNN model was achieved using the Adaptive Moment Estimation (ADAM) training approach with a finalized learning rate of 0.0001, 80 epochs, and an 8-batch batch size. The proposed deep learning model was trained in an impressive average duration of 1024 s. Following a series of experiments, we were able to find the optimal range for each of the training parameters that make up the suggested CNN, which we list in Table 8. Figure 5 represents the confusion matrix derived from simulation results for the recommended deep CNN model with and without data modeling. The confusion matrix that was generated simply contains two classes: diabetes and non-diabetes. Figure 6 depicts the training, validation accuracies, and loss in relation to the number of epochs for diabetes prediction in Fig. 7. Table 9 presents the detailed performance metrics for the deep CNN model with and without a data modeling approach. The results of the suggested deep CNN show considerable performance when compared to the results of standard machine learning classifiers, with a margin of improvement of 16.13%. However, in comparison to the direct evaluation of deep CNN, the data modeling technique that was proposed achieved exceptional results, with an increase in prediction accuracy of 7.75%. In addition, the deep CNN technique with data modeling outperforms all other classification models with an overall accuracy of 96.13%. Figure 8 depicts the corresponding receiver operating characteristic (ROC) curve and precision-recall curve for diabetes prediction utilizing deep CNN in conjunction with a data modeling approach.

**Table 8:** Optimized parameters for hyper-tuning of the customized CNN

**Hyperparameter**	**Assigned value**
Model	Customized CNN
Input	Numerical features
Input size	768x8 (original) 768x32 (data modeling)
Input labels	2 (Diabetic and Normal)
Learning rate	0.0001
Number of epochs	80
Batch Size	8
Optimizer	ADAM
Loss function	Cross entropy
Training time	1024 sec

**Table 9:** Performance evaluation for DL model

**Performance parameter**	**Deep CNN model**	
	**Evaluation without data modeling**	**Evaluation with data modeling**
Accuracy (%)	88.38	96.13
Precision (%)	83.33	94.44
Recall (%)	83.34	94.42
F-Measure (%)	83.33	94.46
Specificity (%)	91.09	97.03
Sensitivity (%)	83.02	94.45
ROC area	0.872	0.959
False Positive Rate (FPR)	0.089	0.029
False Negative Rate (FNR)	0.166	0.055
Matthews Correlation Coefficient (MCC)	0.744	0.914

## Discussion

In this section, the simulated outcomes of the data modeling strategy for diabetes classification have been compared against each other and also with other recent similar studies. It has been observed that the proposed data modeling approach yields significant improvement with the application of either ML or DL models. With the proposed data modeling technique, ML models, specifically the Random Forest classifier, have exhibited increased performance metrics, with an accuracy of 82.82%. A simple deep CNN algorithm proposed for DL models achieved classification accuracies of 88.38% and 96.13% with and without data modeling, respectively. The detailed comparison with recent existing methods is reported in Table 10.

**Table 10:** Comparison with existing methods for diabetes prediction

**Cite**	**Authors**	**Model**	**Techniques**	**Performance parameter**
[48]	Iyer et al. (2015)	ML model with PIMA Indian Diabetes dataset	J48 Naïve Bayes	74.86 % (Accuracy) 79.56 % (Accuracy)
[49]	Mamuda et al. (2017)	ML based Learning algorithms with PIMA Indian Diabetes dataset	Levenberg-Marquardt learning algorithm Bayesian regulation learning algorithm Scaled conjugate gradient learning algorithm	0.00025091 (MSE) 2.021e-05 (MSE) 8.3583 (MSE)
[50]	Kaur et al. (2018)	ML based supervised machine learning algorithm.	Radial Basis Kernel SVM	0.85 (AUC)
[51]	Hang Li et al. (2019)	ML Predictive model	Gradient Boosting Method	0.87 (Recall)
[52]	Soltani et al. (2016)	A new ML based Artificial Neural Network with PIMA Indian Diabetes dataset	Probabilistic Neural Network (PNN)	81.49% (Test accuracy)
	Proposed Technique	ML based Classifiers with data modeling approach on PIMA Indian diabetes dataset	Random Forest (RF)	82.82% (Accuracy) 81.59 (Precision) 81.88 (Recall) 0.855 (ROC)
[53]	Zhou et al. (2020)	DPLD (Deep Learning for Predicting Diabetes) with PIMA Indian Dataset	Enhanced deep neural network with dropout regularization	94.02% (Accuracy)
[54]	Gupta et al. (2021)	ML model and DL Model with PIMA Indian diabetes dataset	QML (Quantum Machine Learning Model) DL network trained with root mean square propagation (RMSprop)	0.85 (Accuracy) 0.74 (Precision) 0.79 (F1 score) 0.95 (Accuracy) 0.90 (Precision) 0.93 (F1 score)
[55]	Krishnamoorthi et al. (2022)	Unique Intelligent Diabetes Mellitus Prediction framework (IDMPF) with PIMA Indian diabetes dataset	Random Forest (RF) Proposed Logistic Regression	81% (Accuracy) 90% (Accuracy)
	Proposed Technique	DL model with PIMA Indian diabetes dataset	7-layered deep convolutional neural network	88.38% (Accuracy) 83.33% (Precision)
	Proposed Technique	Customized deep learning with data modeling approach	7-layered deep convolutional neural network with data modeling	96.13% (Accuracy) 94.44% (Precision) 0.957 (AUC)

### Statistical analysis for proposed data modeling approach

Machine learning relies heavily on statistical analysis, specifically hypothesis testing, to compare various learning methods [56]. Assuming that different classifiers have been evaluated using cross-validation on the same data set, the correlated paired t-test is the method that should be used in order to determine which one is superior [57]. In this work, statistical analysis is used to validate the importance of the data modeling technique that is recommended to be applied to various classifiers. A paired t-test is conducted on the same classifier with and without data modeling to examine the statistical significance of the difference between the two approaches. In order to apply paired t-test, 5-fold cross-validation on a single dataset is used to compute different test accuracies. The parameters of the suggested paired t-test were computed Eqs. (10)–(13) as follows:

15 $$\begin{aligned} {D_i}(A)\, = \,Acc(CF) - Acc(C{F_{DM}}) \end{aligned}$$

where $D_i$ (A) is the differences in accuracies, Acc(CF) is the accuracy of the classifier, and Acc($CF_DM$) is the accuracy of the classifier with data modeling.

16 $$\begin{aligned} m= & {} \frac{1}{n}\sum \limits _{i = 1}^n {{D_i}(A)} \end{aligned}$$

17 $$\begin{aligned} {\sigma _{diff}}= & {} \sqrt{\frac{{\sum \limits _{i = 1}^n {({D_i}(A)} - m{)^2}}}{{n - 1}}} \end{aligned}$$

18 $$\begin{aligned} {t_{statistic}}= & {} \frac{{m \times \sqrt{n} }}{{{\sigma _{diff}}}} \end{aligned}$$

Where m is the mean of the difference between the accuracies and n is the number of observations (5-fold CV), $\sigma _diff$ is the Standard deviation of the differences of the accuracies of the classifier with and without data modeling. The essential parameters for conducting a paired t-test to determine statistical significance are presented in Table 11. In each test, the same classifier is tested with and without a data modeling (DM) approach and calculated t-statistic to know the significance of the proposed technique. For maintaining good practice in statistics, the level of significance is set to be 5% ($\alpha$=0.05). In this study, we compared the computed value of the t-statistic with the *t*-critical value, which is determined by the degrees of freedom (*n* − 1) and the level of significance (5%). Because the t-statistic in each instance is higher than the *t*-critical value, the proposed data modeling approach can be considered statistically significant and is hence acceptable.

**Table 11:** Optimized parameters for hyper-tuning of the Customized CNN

**Test Name**	*** m***	$\varvec\sigma _{\bf diff}$	**t**_**static**_	**t**_**critical**_	**Significance**
MLP-NN Vs MLP-NN-DM	4.020	0.967	4.1565	2.776	Yes
SVM Vs SVM-DM	3.284	0.379	8.6645	2.776	Yes
RF Vs RF-DM	5.820	0.491	11.8450	2.776	Yes
Deep CNN Vs Deep CNN-DM	6.861	0.393	17.4469	2.776	Yes

## Conclusion

As previously mentioned, a significant portion of the human population is affected by diabetes. If left unchecked, it will pose a grave threat to the global community. Therefore, in our proposed research, we designed a robust diabetic prediction model by combining a data modeling approach with ML and DL algorithms. Moreover, the significance of pre-processing has been examined, and it has been determined that it plays a crucial role in accurate and reliable prediction. However, the suggested research primarily focused on establishing a data modeling framework with the goal of providing more relevant data to the learning algorithm’s input in order to improve accurate diabetes prediction among individuals. PIMA Indian Diabetes (PID) data from the UCI machine learning repository database was used in the experiment. During each test, both the original input dataset and the suggested redesigned dataset were used to validate the performance of the classification algorithms. Compared to the original dataset, it has been observed that the input dataset using the data modeling technique significantly improves the performance of the recommended ML models. Furthermore, the proposed data modeling framework was also applied to a seven-layered deep CNN model and achieved promising accuracy of 96.13% for early prediction of diabetes. Overall, the proposed data modeling strategy enhanced the accuracy of all the suggested ML and DL models by an average of 10%. In the future, we plan to create a comprehensive system in the form of a website or mobile application that uses the proposed data modeling approach to assist healthcare professionals in the early detection of diabetes.

## Data Availability

The dataset analysed in the current study is a publicly available open-source PIMA Indian dataset from the UCI Machine Learning repository https://www.kaggle.com/datasets/uciml/pima-indians-diabetes-database.
